# LncRNA PCIR Is an Oncogenic Driver *via* Strengthen the Binding of TAB3 and PABPC4 in Triple Negative Breast Cancer

**DOI:** 10.3389/fonc.2021.630300

**Published:** 2021-05-03

**Authors:** Wenhui Guo, Jingyi Li, Haobo Huang, Fangmeng Fu, Yuxiang Lin, Chuan Wang

**Affiliations:** ^1^ Department of Breast Surgery, Fujian Medical University Union Hospital, Fuzhou, China; ^2^ Department of General Surgery, Fujian Medical University Union Hospital, Fuzhou, China; ^3^ Breast Cancer Institute, Fujian Medical University, Fuzhou, China; ^4^ College of Integrated Traditional Chinese and Western Medicine, Fujian University of Traditional Chinese Medicine, Fuzhou, China; ^5^ Department of Blood Transfusion, Fujian Medical University Union Hospital, Fuzhou, China

**Keywords:** long non-coding RNA, RP11-214F16.8/Lnc-PCIR, TNF-α/NF-κB signaling pathway, TGF-beta activated kinase 1 (MAP3K7) binding protein 3, poly(A) binding protein cytoplasmic 4

## Abstract

Long non-coding RNAs (LncRNA) as the key regulators in all stages of tumorigenesis and metastasis. However, the underlying mechanisms are largely unknown. Here, we report a lncRNA RP11-214F16.8, which renamed Lnc-PCIR, is upregulated and higher RNA level of Lnc-PCIR was positively correlated to the poor survival of patients with triple negative breast cancer (TNBC) tissues. Lnc-PCIR overexpression significantly promoted cell proliferation, migration, and invasion *in vitro* and *in vivo*. RNA pulldown, RNA immunoprecipitation (RIP) and RNA transcriptome sequencing technology (RNA-seq) was performed to identify the associated proteins and related signaling pathways. Mechanistically, higher Lnc-PCIR level of blocks PABPC4 proteasome-dependent ubiquitination degradation; stable and highly expressed PABPC4 can further increase the stability of TAB3 mRNA, meanwhile, overexpression of Lnc-PCIR can disrupt the binding status of TAB3 and TAB2 which lead to activate the TNF-α/NF-κB pathway in TNBC cells. Our findings suggest that Lnc-PCIR promotes tumor growth and metastasis *via* up-regulating the mRNA/protein level of TAB3 and PABPC4, activating TNF-α/NF-κB signaling pathway in TNBC.

## Background

Breast cancer is the most frequently diagnosed malignancy in women worldwide and is the second leading cause of cancer-related death in the United States (1, 2). Expression of the estrogen receptor (ER), progesterone receptor (PR), and amplification of the HER2 gene define the main breast cancer subtypes in terms of prognostic and therapeutic intervention (3). Triple negative breast cancer (TNBC) is the breast cancer subtype characterized by the absence of expression of the ER, PR and HER2 (4).Therapies commonly used in other breast cancer subtypes are therefore not suitable for TNBC, and treatment options are largely limited to conventional genotoxic chemotherapy (5, 6). Most of TNBC patients present high rates of metastatic recurrence and very poor long-term prognosis after chemotherapy. Consequently, to explore the underlying mechanisms and identification of molecular targets and the development of new therapeutic avenues remain critically important.

Long non-coding RNAs (lncRNAs) are a class of transcripts longer than 200 nucleotides lacking the open reading frame with no protein-coding ability (7). With advancements in cancer transcriptome profiling, large number of lncRNAs has been demonstrated closely associated with cancer (8, 9). Besides, lncRNAs have also been implicated to regulate a range of biological functions, such as genomic imprinting and transcriptional regulation, plays a critical role in tumorigenesis and metastasis (10–12). During the past decade, numerous studies have showed the important role of NF-κB pathway as a link between inflammation and tumorigenesis (13). NF-κB was first identified as part of the immune system, and become widely accepted as a crucial transcription factor that regulates inflammation, innate and adaptive immunity, cell proliferation, cell differentiation and apoptosis (14, 15). The hyper-activate of NF-κB pathway has been linked to cancer which is regarded as a potential therapeutic target in human cancers (16, 17). TGF-beta activated kinase 1 (MAP3K7) binding protein 3 (TAB3), as a newly identified Transforming growth factor-β-activated kinase 1 (TAK1) binding partner, has been implicated in the immune response, signal transduction, inflammation and autophagy (18–21). Several reports showed TAB3 is markedly overexpressed in various tumor tissues, such as the testis, skin, non-small cell lung cancer (NSCLC), hepatocellular carcinoma (HCC) and small intestinal cancers (19, 22–24). However, the mechanism of overexpressed TAB3 in the process of TNBC remains unclear.

In this study, we sought to identify clinically relevant lncRNAs deregulated specifically in TNBC patients and aim to reveal the functional role and regulatory mechanism of lncRNA in the progress of TNBC. We identified and characterized the lncRNA RP11-214F16.8, renamed Lnc-PCIR (LncRNA Positively Correlated with Inflammatory Responses). Our results showed Lnc-PCIR was upregulated in TNBC tissues, and overexpressed of Lnc-PCIR could accelerated cell growth and metastasis *in vitro* and *in vivo*. For mechanistic investigations, Lnc-PCIR could directly binding with TAB3 and PABPC4. The stability of PABPC4 protein was increased by inhibiting ubiquitin proteasome degradation and overexpressed PABPC4 can enhance the stability of TAB3 mRNA, which known as a key molecular involved in TNF-α/NF-κB signaling pathway. Taken together, these results suggest that Lnc-PCIR may be as the promising therapeutic target for TNBC patients.

## Methods

### Cell Culture

Human TNBC cell lines used in this study were purchased from Cell Bank of the Chinese Academy of Science (Shanghai, China), the Health Science Research Resources Bank (Osaka, Japan) and American Type Culture Collection (ATCC, Manassas, Virginia, USA). The cell lines were cultured in Dulbecco’s modified Eagle’s medium (DMEM, Gibco BRL) contained with 10% fetal calf serum (FBS, HyClone) as well as 100 U/ml penicillin and 100 μg/ml streptomycin (Invitrogen). Cells were maintained in a humidified incubator at 37°C in the presence of 5% CO2. Cell lines confirmed to be mycoplasma-free by short tandem repeats (STR) profiling. All the cell lines were used within 15 passages and subjected to routine cell line quality examinations (e.g., morphology, mycoplasma), and thawed fresh every 2 months.

### Patients and Samples

Cohort 1: One hundred and ten paired TNBC and neighboring noncancerous tissues from TCGA database (https://cancergenome.nih.gov/); Cohort 2: Five hundred and fifty paired patients TNBC and neighboring noncancerous tissues were also from TCGA database; Cohort 3: One hundred and ten paired TNBC and neighboring noncancerous tissues obtained from the surgical specimen archives of the Shanghai cancer center of Fudan university, Shanghai, China (collected postoperatively from August 2010 to September 2018); Each sample was snap-frozen in liquid nitrogen and stored at −80°C prior to RNA isolation and qRT-PCR analysis. All patients recruited to this study did not receive any pre-operative treatments. The data do not contain any information that could identify the patients. All patients provided written informed consent. The use of human clinical specimens in the present study was approved by the Institutional Review Board of the Shanghai Medical College of Fudan University. A summary of the clinical information for the 110 patients is available online in **Supplementary Table 1**.

### RNA Isolation and Quantitative PCR (Real-Time RT-PCR)

Total RNA was extracted from tissues or cultured cells using TRIzol reagent (Invitrogen). Total RNA (500 ng) was reverse transcribed to cDNA in a final volume of 10 μl using random primers under standard conditions with the PrimeScript RT Reagent Kit (Takara, Dalian, China). We performed real-time PCR analyses using SYBR Premix Ex Taq (Takara) according to the manufacturer’s instructions. Results were normalized to the expression of glyceraldehyde 3-phosphate dehydrogenase (GAPDH) or β-actin, and data were collected based on the comparative cycle threshold (CT) (2^−ΔΔCT^) method. Specific primer sequences are listed in **Supplementary Table 2**.

### Plasmid Generation and Transfection/Infection

We obtained the full-length Lnc-PCIR, PABPC4 and TAB3 sequence and ligated into the pcDNA3.0 (+) vector (Invitrogen). Additionally, we also cloned the sequence of PABPC4 and TAB3 into the pCMV-Flag or pCMV-HA. Plasmid were transfected into TNBC cells cultured in six-well plates using the Lipofectamine 3000 DNA transfection reagent (life, USA). For lentivirus packaging, 2 × 10^6^ 293 T cells were co-transfected with packaging plasmid pA2 and pMD2G. After 8 h, the transfection medium was replaced with fresh DMEM supplemented with 10% FBS. Subsequently, cell supernatants containing lentiviruses were collected. For infection, cells were treated with polybrene (8 μg/mL, Sigma) for 0.5 h before incubated with lentiviral, after incubation for 8 to 12 h at 37°C, stable cell lines were selected by puromycin (2 μg/mL, Sigma). Cells were harvested for qRT-PCR or western blot analysis 48 h after transfection. The sequences for the gene-specific primers used are listed in **Supplementary Table 2**.

### Northern Blot Assay

Total RNA (10–15 μg) from samples were separated on 15% denaturing polyacrylamide gels, transferred onto GeneScreen Plus membranes (PerkinElmer), and hybridized using UltraHyb-Oligo buffer (Ambion). Following hybridization at 42°C overnight, the membranes washed twice in 0.1×SSPE and 0.1% SDS at 42°C for 15 min each. Membranes were then exposed to a storage phosphor screen (GE Healthcare Bio-Sciences) for 8 h and imaged using a Typhoon 9410 Variable Mode Imager (GE Healthcare Bio-Sciences). The sequences for probe primers used are listed in **Supplementary Table 2**.

### Western Blotting

Cells were lysed in lysis buffer in the presence of protease inhibitor cocktail (Roche) and phosphatase inhibitor cocktails I and II (Sigma). Equal amounts of protein, as determined by the Bradford assay, were resolved by electrophoresis in a SDS 10% polyacrylamide gel and then transferred to a PVDF membrane (Millipore). The membrane was incubated with the primary antibodies and secondary antibodies, then was detected using an enhanced chemiluminescence kit (ECL, Invitrogen). The details of antibodies used are listed in **Supplementary Table 3**.

### Cell Growth Assay

For cell growth assays, 2000 cells per well were seeded into 96-well plates, with three wells used for each group. Cell numbers were evaluated for 5 days using a cell counting kit-8 (CCK-8) (Dojindo, Japan). 10 μl of CCK-8 reagent was added to each well and the plate was incubated at 37°C for 2 h. Next, the absorbance at 450 nm was measured in each well by using a spectrophotometer (Molecular Devices, CA, USA). For the colony formation assay, 1500 cells well were seed into 6-well plates and routinely cultured for 14 days. The cells were subsequently fixed with 30% formaldehyde for 10 min and stained with 0.1% crystal violet for 10 min. The number of colonies was determined under an optical microscope.

### Cell Migration and Invasion Assays

The migration and invasive ability of the cells was performed using a transwell assay. For Invasion assays, we using the 8-μm pore inserts Millicell chambers which were coated with 30 μg of Matrigel (BD Biosciences, USA). And the Millicell chambers without coating the Matrigel was conducted in the migration assay. Cells (5×10 (4) for migration and 1×10 (5) for invasion) were seeded onto a transwell plate with 8-mm pores, and DMEM supplemented with 20% FBS was used as a chemoattractant. Following a 24-h incubation, non-invading cells were manually removed using a cotton swab. Subsequently, the cells were fixed in 4% paraformaldehyde for 20 min, stained with hematoxylin and then counted under a microscope.

### RNA Pull-Down and Mass Spectrometry Assay

A full-length of sense and antisense Lnc-PCIR sequence were transcribed and biotin-labeled using T7 RNA polymerase (Roche, Basel, Switzerland) and purified using the RNA Clean Kit (ZYMO research, USA) *in vitro*. 1 mg protein lysis of 231 cell extracts was then mixed with 2 μg of biotinylated RNA at 4°C for 1 h, and then incubated with 50 μl of M-280 streptavidin dyna-beads (Thermo Scientific, USA) over night at 4°C. After washing with the beads for six times with DEPC-PBS buffer. The RNA–protein complex was boiled in 1×SDS buffer for 5 min. The retrieved protein was detected using standard western blotting techniques and silver staining.

For mass spectrometry process, in brief, which include protein digestion, MS, database retrieval and protein identification. Firstly, protein digestion, each sample was allowed to proceed at 56°C for 1 h in 10 Mm dithiotreitol and add 55 mM iodoacetamide incubated in the dark for 45 min at room temperature. The gel pieces were washed with 100 μl of 25 mm NH4HCO3 for 10 min and dehydrated with 100 μl of 25 mm NH4HCO3 in 50% acetonitrile for 5 min for two times. Following drying in a SpeedVac, the gel pieces were mixed with 12.5 ng/μl of trypsin and incubated on ice for 40 min and 25 mm NH4HCO3 was added as needed to cover the gel pieces. Digestion was then carried out at 37°C overnight. Using 60% acetonitrile, 0.2% TFA to extract the tryptic peptides from the gel pieces. Following 20 min of vortex and 5 min of sonication, the supernatant was taken and saved. Following the evaporation of acetonitrile in a SpeedVac, the sample was desalted with a C18 ZipTip (Millipore), and half of the eluate was analyzed with nanoLC-MS/MS. Secondly, the samples were resuspended with 30 μl solvent C respectively (C: water with 0.1% formic acid), separated by nanoLC and analyzed by on-line electrospray tandem mass spectrometry. 10 μl peptide sample was loaded onto the trap column (Thermo Scientific Acclaim PepMap C18, 100 μm × 2 cm), with a flow of 10 μl/min for 3 min and subsequently separated on the analytical column (Acclaim PepMap C18, 75 μm × 15 cm) with a 90-min linear gradient, from 5% D (D: ACN with 0.1% formic acid) to 55% D. The column was re-equilibrated at initial conditions for 10 min. The column flow rate was maintained at 300 nl/min. The electrospray voltage of 2 kV versus the inlet of the mass spectrometer was used. Thirdly, tandem mass spectra were processed by PEAKS Studio version 8.5 (Bioinformatics Solutions Inc., Waterloo, Canada). PEAKS DB was set up to search the UniProt-homo sapiens database (version 201712, 72029 entries) assuming trypsin as the digestion enzyme. Peptides were filter by 1% FDR and 1 unique peptide.

### RNA Immunoprecipitation (RIP) Assay

RIP was performed using the EZ-Magna RIP kit (Millipore, Billerica, MA) following the manufacturer’s protocol. 231 cells at 80–90% confluency was scraped off the tissue culture plate, then lysed in complete RIP lysis buffer. A total of 200 μl of whole cell extract was incubated with RIP buffer containing magnetic beads conjugated with antibodies against PABPC4 and TAB3 or control IgG (Millipore) overnight at 4°C. The beads were washed with wash buffer, then the complexes were incubated with 0.1% SDS and 0.5 mg/ml Proteinase K (30 min at 55°C) to remove proteins. The RNA concentration was measured using a NanoDrop spectrophotometer (Thermo Scientific, USA) and its quality was assessed using a bioanalyzer (Agilent, Santa Clara, CA). Finally, immunoprecipitated RNA was purified and analyzed by qRT-PCR.

### Subcellular Fractionation

The separation of nuclear and cytosolic fractions was performed using the NE-PER Nuclear and Cytoplasmic Extraction Reagents Kit (Thermo, USA) following the manufacturer’s instructions. In brief, for adherent cells, harvest with trypsin-EDTA and then centrifuge at 500 × g for 5 min; Wash cells by suspending the cell pellet with PBS and centrifuge at 500 × g for 2 min for two times; Add ice-cold 200 μl CER I to the cell pellet, and vortex the tube vigorously on the highest setting for 15 s to fully suspend the cell pellet. Incubate the tube on ice for 10 min, add ice-cold 11 μl CER II, vortex the tube for 5 s on the highest setting. Incubate tube on ice for 1 min; Centrifuge the tube for 5 min at 16,000*g*, transfer the supernatant (cytoplasmic extract) to a tube; suspend the pellet with 200 μl ice-cold NER, vortex on the highest setting for 15 s every 10 min, for a total of 40 min; Centrifuge the tube at 16,000 × g for 10 min; Immediately transfer the supernatant (nuclear extract) fraction to a clean pre-chilled tube, store extracts at −80°C until use. RNA isolation using TRIzol reagent (Invitrogen) and using PrimeScript RT Reagent Kit (Takara, Dalian, China) for cDNA production. β-Actin was used as the cytoplasmic endogenous control. U6 small nuclear RNA was used as the nuclear endogenous control for qPCR.

### RACE Assay

We used a SMARTer RACE cDNA Amplification kit (Clontech, California, USA) to determine the transcriptional initiation and termination sites of Lnc-PCIR, according to manufacturer’s instructions. The sequences for the gene-specific PCR primers used for 5’ and 3’ RACE analysis was given in **Supplementary Table 2**.

### RNA Scope Assay

RNA *in situ* hybridization was performed using RNAScope^®^ Multiplex Reagent Kit for Tissues (ACD, Life technologies, USA) to analysis the RNA level of Lnc-PCIR in human TNBC tissues and adjacent normal tissues. In brief, deparaffinized tissue sections were hybridized with the Lnc-PCIR probe and negative control probe at 40°C for 2 h. After hybridizations, sections were subjected to signal amplification, Gill’s hematoxylin counterstaining, and scanning (Aperio ScanScope CS, Leica Biosystems, Nussloch, Germany) at 40× magnification. Fast Red semiquantitative image analysis was performed using the Aperio RNA ISH algorithm, which automatically quantifies the staining across whole slides and counts individual molecular signals and clusters in the cells. The obtained results are divided into three ranges: 1, which includes cells containing two to five dots per cell; 2, which includes cells containing 6-20 dots per cell; and 3, which includes cells containing more than 20 dots per cell.

### Tumor Xenograft Experiments

Female NOD/SCID nude mice (4 weeks old) were obtained from the Shanghai Model Organisms Center (Shanghai, China). And housed and maintained in laminar airflow cabinets under specific pathogen-free conditions. Subsequently, the stable lenti-P-Lnc-PCIR or control 231 cells (1 × 10^7^ cells/mice in 200 μl sterile PBS) were injected subcutaneously into NOD/SCID nude mice. Tumor growth was measured after 1 week, and tumor volumes were calculated by the formula: volume (cm^3^) = (length × width^2^)/2. After 4 weeks, the mice were sacrificed and the tumors were collected and weighed. For *in vivo* metastasis assay, 1 × 10^6^ lenti-P-Lnc-PCIR or control 231 cells (in 50 μl of sterile PBS) were orthotopically injected directly into the inguinal mammary fat pads of mice in (n = 10 in each group). All procedures were conducted in accordance with the Guidelines for the Care and Use of Laboratory Animals with the approval of the Ethics Committee of the Fujian Medical University.

### Statistical Analysis

All experiments were performed in triplicate. Statistical analyses were performed using SPSS (version 23.0, SPSS Inc.) or GraphPad Prism software (version 7.0, USA). Clinicopathological characteristics were analyzed by chi-square tests. Survival curves were generated using the Kaplan-Meier method and log-rank tests. Univariate and multivariate Cox regression analyses were conducted to identify the independent factors. Student’s t-test or the Mann–Whitney U test was used for comparison between two groups depending on distribution. P (two-sided) less than 0.05 was considered to indicate statistical significance. All data were presented as the mean ± standard error of the mean (SEM).

## Results

### Identification of Clinically Relevant lncRNAs Overexpressed in TNBC

**Figure 1:**
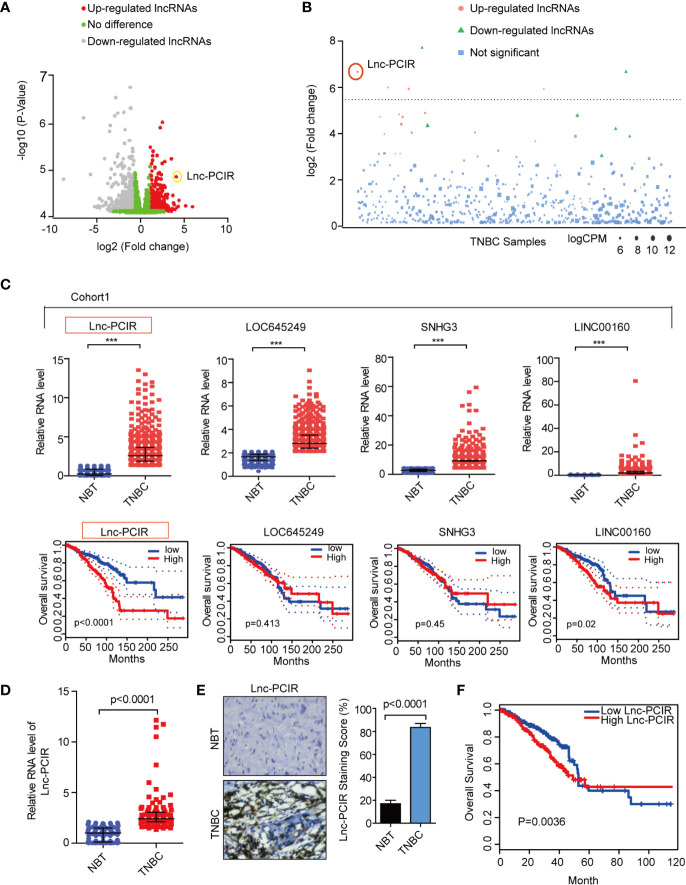
Lnc-PCIR upregulated and predicted the worse survival in triple negative breast cancer. **(A, B)** Volcano plot and Manhattan plot analysis of RNA-sequencing data of TNBC and normal tissues from TCGA. Red represents up-regulated long non-coding RNAs with ${\text{log}}_{2}^{\left(\text{fold change}\right)}$ >4; **(C)** Relative expression level and overall survival of screened lncRNAs in TCGA data of TNBC samples; **(D)** qRT-PCR results showed that Lnc-PCIR expression was significantly upregulated in 110 pairs of TNBC tissues and non-tumor tissues (NTs); **(E)** RNA Scope assay to detect the Lnc-PCIR RNA level in TNBC and adjacent normal tissues. Left panel: representative images; right panel: statistical analysis of the staining; **(F)** Kaplan-Meier survival analysis of Lnc-PCIR expression in TNBC patients (n = 110). All *p*-values calculated by independent sample t-test (***significant values of <0.001).

In order to identify lncRNAs that play a role in TNBC, we used RNA-sequencing (RNA-seq) data from 1084 patients available in the TCGA database (The Cancer Genome Atlas). We classified the tumors with available PAM50 (Prediction Analysis of Microarray 50) molecular subtype annotation (25), obtaining a final cohort of 110 TNBC patients (cohort 1) (26). Using differentially expressed gene analysis, we identified a subset of lncRNAs overexpressed with clinically relevant in the TNBC subtype (fold change>4) compared to normal tissue (**Figures 1A, B** and **Supplementary Excel 1**). Subsequently, the top four upregulated lncRNAs are selected: RP11-214F16.8 (Lnc-PCIR), LOC645249, SNHG3, and LINC00160. The RNA level of Lnc-PCIR was confirmed in 550 paired TNBC tissues and paired tumor-adjacent non-tumor tissues (Cohort2) (**Figure 1C**). The univariate Cox proportional hazards regression method revealed that Lnc-PCIR showed the significantly prognostic value in TNBC tissue (**Figure 1C**). So, we choose the Lnc-PCIR for further study. Moreover, the RNA levels of Lnc-PCIR were confirmed by quantitative real-time polymerase chain reaction (q-PCR) analysis in 110 paired TNBC tissues and paired tumor-adjacent non-tumor tissues (Cohort3). Compared with matched normal tissues, Lnc-PCIR was significantly up-regulated in TNBC tissues (**Figure 1D**). We also employed the RNA Scope assay to analyze Lnc-PCIR RNA level, results confirmed Lnc-PCIR has significantly higher level in TNBC tissues (**Figure 1E**). Patients with higher levels of the Lnc-PCIR exhibited poor survival outcomes (*P*=0.0036) (**Figure 1F**). These data together indicated that Lnc-PCIR was significantly upregulated and related to the overall survival in TNBC tissues, and might be involved in the progression of TNBC.

### Overexpressed Lnc-PCIR Promoted Cell Migration, Invasion, and Proliferation *In Vitro* and *In Vivo*

We first analyzed the basic characteristic of Lnc-PCIR in breast cancer cells. Lnc-PCIR (ENSG00000280710.2) located on 13q32.3 and has only one transcript with three exons (**Supplementary Figure 1A**). Lnc-PCIR is widely expressed in different breast cancer cell lines, and the RNA level was significantly up-regulated in TNBC cell lines (**Supplementary Figure 1B**). Next, we examined the subcellular localization of Lnc-PCIR, finding that Lnc-PCIR predominately resides in the nucleus in 231 and BT549 cells by qRT-PCR (**Supplementary Figure 1C**). By Coding Potential Calculator (27) (http://cpc.cbi.pku.edu.cn/) and the PhyloCSF codon substitution frequency analysis (28), Lnc-PCIR has low protein-coding ability (**Supplementary Figures 1D, E**). Moreover, we performed the RACE assay (rapid amplification of cDNA ends) and Northern blot assay to confirm the Lnc-PCIR is a 987-bp-long intergenic non-protein-coding RNA in breast cancer cells (**Supplementary Figures 1F, G**).

**Figure 2:**
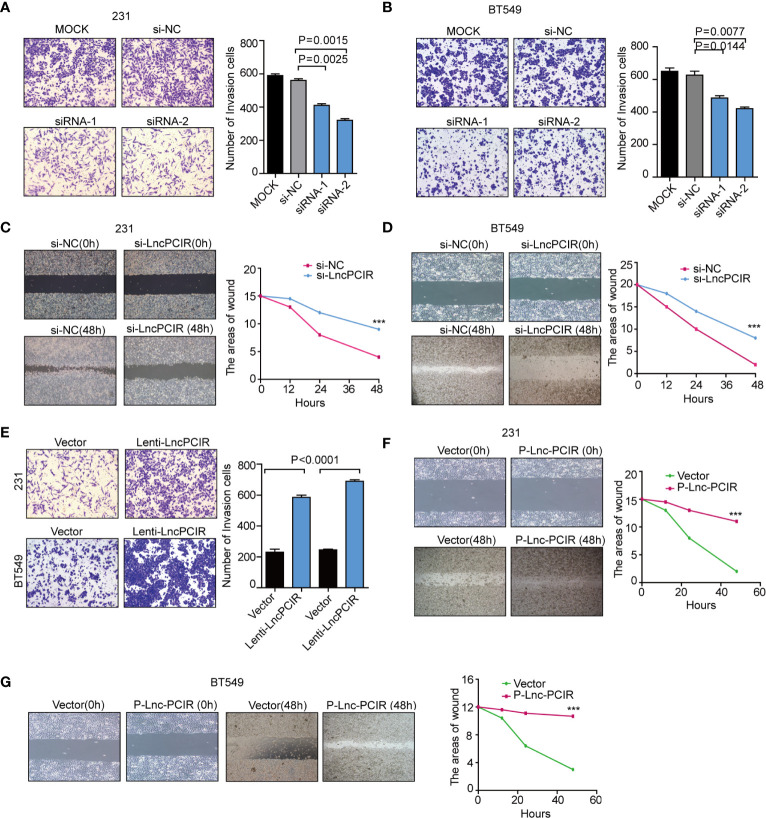
Lnc-PCIR increases TNBC cell invasion and migration *in vitro*.**(A, B)** Transwell invasion assays in the 231 **(A)** and BT549 **(A)** cells with Lnc-PCIR knockdown; **(C, D)** Wound-healing assays in the 231 **(A)** and BT549 **(A)** cells with Lnc-PCIR knockdown; **(E)** Transwell invasion assays in the 231 **(A)** and BT549 **(A)** cells with Lnc-PCIR overexpressed; **(F, G)** Wound-healing assays in the 231 **(A)** and BT549 **(A)** cells with Lnc-PCIR overexpressed. All *p*-values calculated by independent sample t-test (***significant values of <0.001).

**Figure 3:**
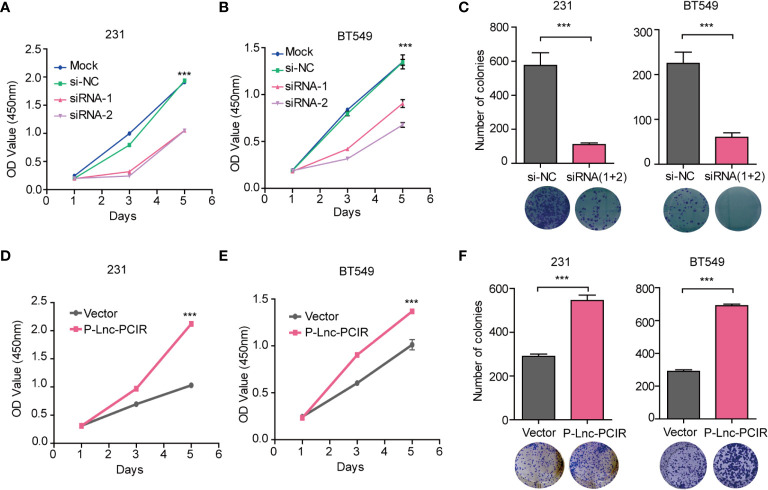
Lnc-PCIR increases TNBC cell proliferation and colony formation *in vitro.* **(A–C)**. CCK-8 assays **(A, B)** and Colony formation assays **(C)** in the 231 and BT549 cells with knockdown of Lnc-PCIR; **(D–F)**. CCK-8 assays **(D, E)** and Colony formation assays **(F)** in the 231 and BT549 cells with overexpression of Lnc-PCIR. All *p*-values calculated by independent sample t-test (***significant values of <0.001).

To assess the effect of Lnc-PCIR on TNBC cell migration and invasion, we performed transwell assay and wound-healing assay. Two independent small interfering RNAs (siRNAs) for Lnc-PCIR significantly decreased the migration and invasion of the 231 and BT549 cells (**Figures 2A–D** and **Supplementary Figure 2A**), and vice-versa, Lnc-PCIR overexpression by a lentivirus vector (pCDH- Lnc-PCIR, shorted in P-Lnc-PCIR) promoted 231 and BT549 cells the migration and invasion of the 231 and BT549 cells (**Figures 2E–G** and **Supplementary Figure 2B**). On the other hand, Lnc-PCIR knockdown significantly decreased 231 and BT549 cells growth and colony formation (**Figures 3A–C**), whereas, Lnc-PCIR induction increased cell growth and colony formation (**Figures 3D–F**).

**Figure 4:**
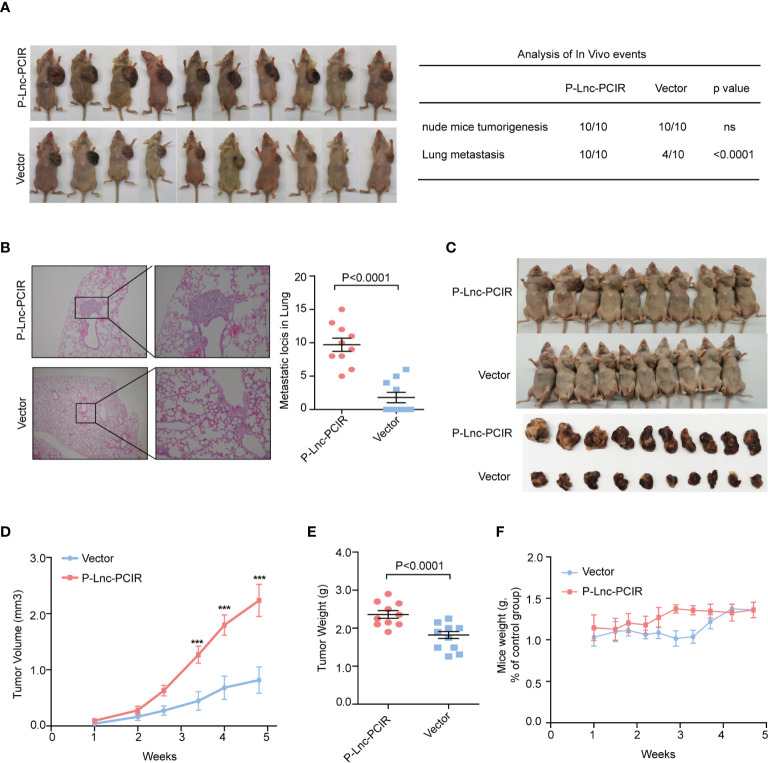
Lnc-PCIR increases TNBC cell metastasis and cell growth *in vivo*.**(A, B)** Statistics analysis of the metastatic foci in the lung obtained from nude mice injection with the stable P-Lnc-PCIR 231 cells detected by hematoxylin-eosin staining; **(C–E)**. Representative image of nude mouse models bearing subcutaneous tumor xenografts **(C)**; Tumor volumes **(D)** and tumor weights **(E)** were measured in the P-Lnc-PCIR 231 cells and negative-control groups in the xenograft mouse models; **(F)** The weight of nude mice in the P-Lnc-PCIR 231 cells group and negative-control group. All *p*-values calculated by independent sample t-test (***significant values of <0.001).

To further explore the growth-promoting effects of Lnc-PCIR on TNBC cells *in vivo*, we evaluated the promoting effects of Lnc-PCIR on cell metastasis. The stable P-Lnc-PCIR 231 cells were transplanted into the fat pad of nude mice. The metastatic nodules in the lung were significantly increased in the P-Lnc-PCIR group (**Figure 4A**). Hematoxylin-eosin staining showed that the metastatic foci derived from the P-Lnc-PCIR cells dramatically increased in the lung (**Figure 4B**). We also subcutaneously injected stable P-Lnc-PCIR 231 cells into nude mice. Both the volumes and weights of the tumors in the P-Lnc-PCIR group were markedly higher than those in the control group (**Figures 4C–E**) with no significant change in body weight of nude mice (**Figure 4F**), demonstrating that P-Lnc-PCIR promotes the tumorigenicity of the TNBC cells *in vivo*. Taken together, these findings suggest that Lnc-PCIR acts as an oncogenic driver in the development and progression of TNBC.

### Identified the Lnc-PCIR Binding Proteins

**Figure 5:**
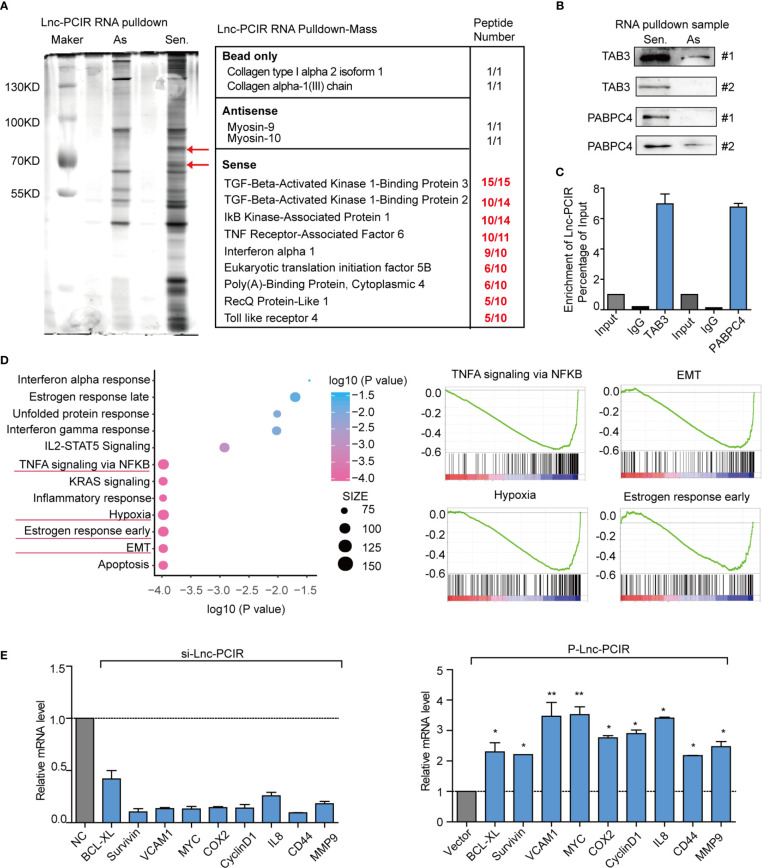
Lnc-PCIR specific directly binding with TAB3 and PABPC4 in 231 cells. **(A)** Silver staining of SDS-PAGE gel of biotinylated Lnc-PCIR RNA pull-down assays. Red arrows indicated the Lnc-PCIR-Sense-specific bands; **(B)** Western blot to analysis the interaction partners of Lnc-PCIR using RNA pull-down samples in 231 cell lines; **(C)** RIP analyses were performed using antibodies against endogenous TAB3 and PABPC4, with IgG as a negative control. The RNA level of the Lnc-PCIR was detected using RT-PCR and normalized to the input; **(D)** GSEA analysis of RNA-sequencing primary data when knockdown of Lnc-PCIR by two independent siRNAs and representative image of top four signaling pathways showed by GSEA analysis; **(E)** Top key genes regulated by TNF-α/NF-κB pathway were verified by qRT-PCR when knockdown or overexpressed Lnc-PCIR in TNBC cells. All *p*-values calculated by independent sample t-test (*significant values of <0.05; **significant values of <0.01).

To explore the molecular mechanism underlying the oncogenic activity of Lnc-PCIR in TNBC progression, we performed RNA pull-down assays to identify the proteins associated with Lnc-PCIR in the 231 cells. The results from three independent Lnc-PCIR pull-down experiments repeatedly showed specific bands at approximately 70 KD and 90 KD *via* mass spectrometry (**Figure 5A** and **Supplementary Table 4**). Nine potential interacting proteins were obtained based on unique peptide number>5 and peptide number>10 in the three independent experiments and were absent in the corresponding antisense groups (**Figure 5A**). After confirming in two independent experiments, we observed that sense but not antisense Lnc-PCIR, was specifically associated with TGF-beta activated kinase 1 (MAP3K7) binding protein 3 (TAB3) and Poly(A) binding protein cytoplasmic 4 (PABPC4) (**Figure 5B**). Moreover, RIP assays showed that the antibodies of TAB3 or PABPC4 could significantly enrich Lnc-PCIR (**Figure 5C**), whereas the GAPDH antibody and IgG as the negative control. In addition, unbiased transcriptome profiling was performed using RNA-sequencing in 231 cells transfected with two independent si-Lnc-PCIR to investigate the related signaling pathways and biological process. GSEA (Gene Set Enrichment Analysis) and GO analysis (Gene Ontology Analysis) showed that top four signaling pathways Lnc-PCIR involved in which include: TNFA signaling *via* NFKB pathway, Hypoxia, Epithelial-mesenchymal Transition (EMT) and Estrogen response early in 231 cells (**Figure 5D** and **Supplementary Excel 2**). Moreover, we verified the top-scoring genes altered in TNFA signaling *via* NFKB pathway and confirmed that Lnc-PCIR dramatically affected the genes expression level that are highly associated with tumorigenesis (**Figure 5E**). Taken together, these findings demonstrated that Lnc-PCIR is an oncogenic driver through activating TNF-α/NF-κB signaling pathway by binding with TAB3 and PABPC4 in TNBC and the molecular mechanisms between them need further explored.

### Lnc-PCIR Enhances the Protein Levels of TAB3 and PABPC4

**Figure 6:**
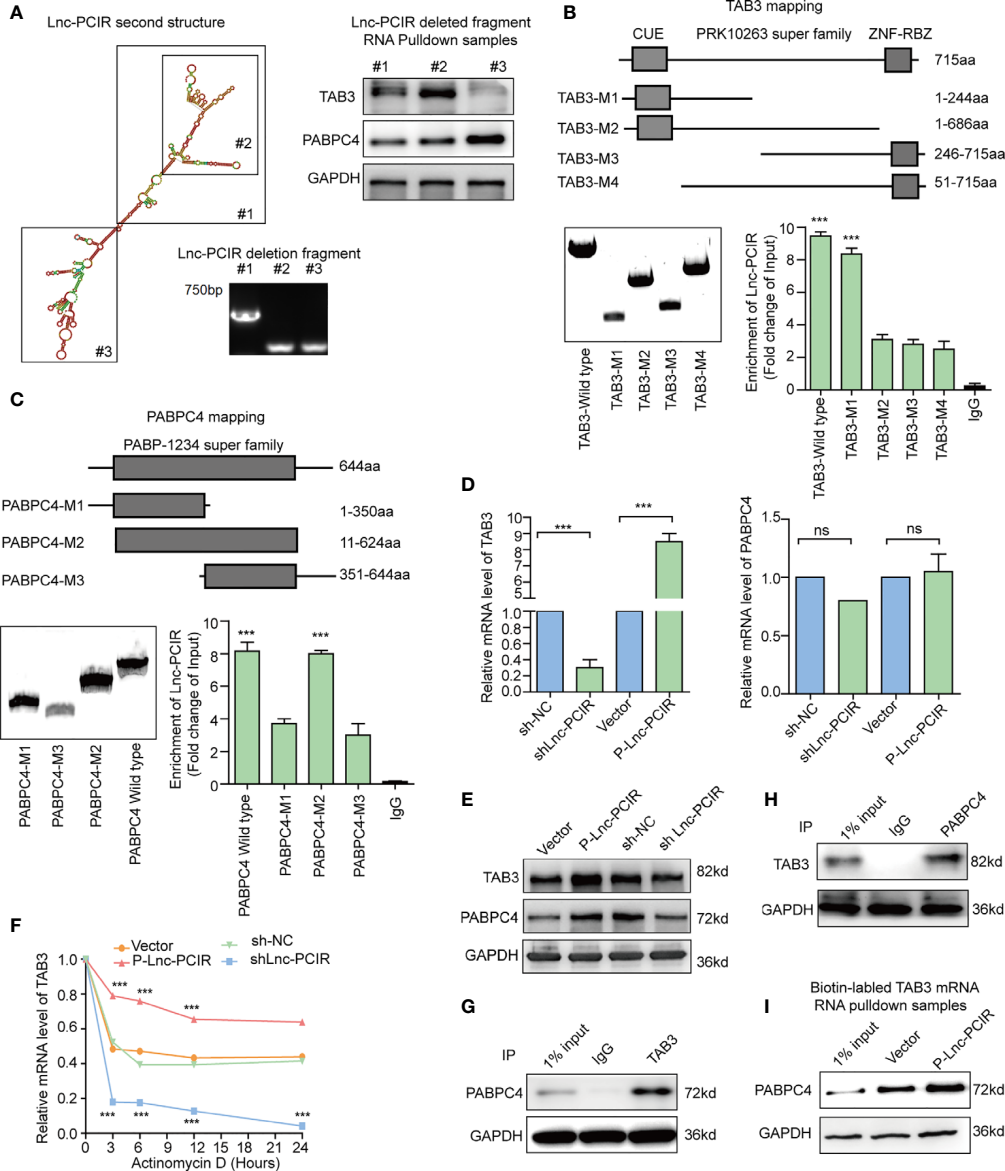
Lnc-PCIR enhances the protein levels of TAB3 and PABPC4. **(A)** Deletion mapping of the Lnc-PCIR according to the second structure (https://lncipedia.org/); Western blot of TAB3 or PABPC4 in pull-down samples by full-length biotinylated-Lnc-PCIR or truncated biotinylated- Lnc-PCIR RNA motifs, with GAPDH as the negative control; **(B, C)** RIP analysis of deletion mapping for the domains of TAB3 **(B)** or PABPC4 **(C)** that bind to Lnc-PCIR; **(D)** The mRNA levels of TAB3 or PABPC4 were quantified by qRT-PCR with Lnc-PCIR knockdown or overexpression; **(E)** The protein levels of TAB3 or PABPC4 after Lnc-PCIR overexpression or knockdown. GAPDH served as the internal control; **(F)** The half-life of TAB3 after treatment with actinomycin D for indicated times, with Lnc-PCIR knockdown or overexpression; **(G, H)** Co-IP assay to detect the association between TAB3 and PABPC4; **(I)** Western blot to analysis the interaction of PABPC4 with biotinylated-TAB3 mRNA using RNA pull-down samples in 231 cell lines. All *p*-values calculated by independent sample t-test (***significant values of <0.001). ns, no significance.

In order to explore the regulatory mechanism among Lnc-PCIR, TAB3 and PABPC4, we first analysis the binding fragment/domain between them, a series of deletion was constructed based on the secondary structure of Lnc-PCIR (http://www.lncipedia.org/, **Figure 6A**). The 201–507 nt (#2) fragment of Lnc-PCIR mediates the interaction with TAB3, while the 625–987 nt (#3) fragment of Lnc-PCIR is required and sufficient for the association with PABPC4. Additionally, we also construct the FLAG-tagged full-length and truncated TAB3 or PABPC4. RIP assays showed CUE domain (1–244 aa) of TAB3 was the vital domain mediate the interaction with Lnc-PCIR (**Figure 6B**), and PABP-1234 super family domain (11–624 aa) of PABPC4 is required for its association with Lnc-PCIR (**Figure 6C**). Next, we sought to determine the functional relevance of the association between Lnc-PCIR and TAB3/PABPC4. Knockdown of Lnc-PCIR significantly decreased the mRNA level of TAB3, and vice versa (**Figure 6D**). But knockdown and overexpressed Lnc-PCIR has non-effect of mRNA level of PABPC4 (**Figure 6D**). Western blot assay revealed overexpressed Lnc-PCIR increased the expression level of TAB3 and PABPC4, knockdown of Lnc-PCIR could significantly reduce the protein level of TAB3/PABPC4 in 231 cells (**Figure 6E**). Moreover, actinomycin D, which effectively inhibits the *de novo* synthesis of RNA, was used to explore the stability of TAB3 regulated by Lnc-PCIR. Overexpression of Lnc-PCIR could increase the half-life and steady-state level of TAB3, whereas the depletion of Lnc-PCIR resulted in a decreased half-life and RNA level of TAB3 (**Figure 6F**), revealing that Lnc-PCIR specifically regulate the stability of TAB3 in the TNBC cells. Meanwhile, we found PABPC4 could specifically bind with TAB3 in 231 cells (**Figures 6G, H** **)**. PABPC4, a Poly (A)-binding protein, is expressed at a higher level in colon cancer and lung adenocarcinoma compared to normal tissues (29, 30). However, the expression profile and role of PABPC4 in TNBC remains unknown. Given this, we speculate whether PABPC4 can directly bind to the TAB3 mRNA. RNA pulldown assay was conducted with biotinylated TAB3 mRNA, results showed overexpressed Lnc-PCIR strengthened the binding of PABPC4 and mRNA of TAB3 (**Figure 6I**). Both PABPC4 and TAB3 are overexpressed in TNBC tissues, and a strong correlation between them (**Supplementary Figures 3A–C**). Furthermore, knockdown of PABPC4 or TAB3 reduced the cell growth and invasion abilities of the 231 cells (**Supplementary Figures 3D, E** and **Supplementary Figures 4A–D**). These findings suggest higher RNA level of Lnc-PCIR could strengthening the stability of TBA3 mRNA, by enhancing the binding state of PABPC4 to TAB3 in TNBC cells.

### Lnc-PCIR Blocks PABPC4 Proteasome-Dependent Ubiquitination Degradation

**Figure 7:**
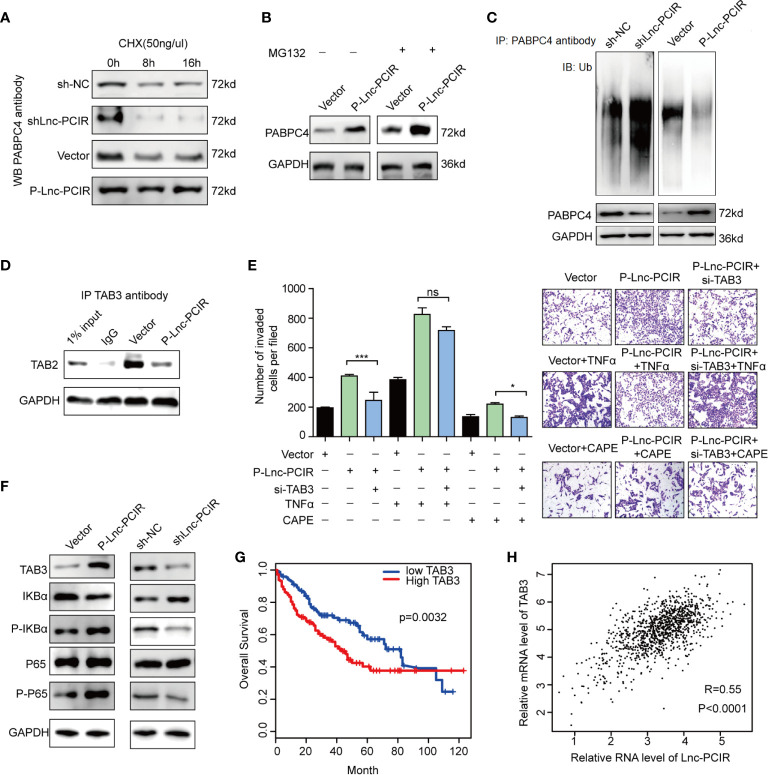
Lnc-PCIR blocks proteasome-dependent ubiquitination degradation of PABPC4. **(A)** Western blot to detect the protein level of PABPC4 in sh-Lnc-PCIR, P-Lnc-PCIR or the control cells which treated with CHX for the indicated times; **(B)** Western blot to detect the protein level of PABPC4 in P-Lnc-PCIR or control cells were treated with MG132 for 12 h; **(C)** IP assay with either control IgG or endogenous PABPC4 antibody and immunoblotted with the ubiquitin-specific antibody, endogenous PABPC4 and GAPDH served as the loading control; **(D)** Co-IP assay to detect the association between TAB3 and TAB2 after Lnc-PCIR overexpression, GAPDH served as the loading control; **(E)** Rescue assays of transwell invasion assay were performed after silencing TAB3 in P-Lnc-PCIR cells with or without TNFα/CAPE treatment; **(F)** Protein expression levels of TAB3, IKBa, p-IKBa, P65 and p-P65 transfected with P-Lnc-PCIR and vector in 231 cells; ** (G)** Kaplan-Meier survival analysis of TAB3 expression in TNBC patients (n = 110); **(H)** Correlation analysis between TAB3 and Lnc-PCIR in TNBC patients (n = 110). All *p*-values calculated by independent sample t-test (**significant values of <0.01). ns, no significance.

Although Lnc-PCIR had no significant effect on the mRNA level of PABPC4 (**Figure 6D**), the protein levels of PABPC4 were dramatically increased when overexpressing Lnc-PCIR and were reduced when silencing Lnc-PCIR in TNBC cells (**Figure 6E**). To explore the mechanisms underlying this phenomenon, we wonder whether it affects the stability of the PABPC4 protein level. Thus, following treatment with a protein-synthesis inhibitor cycloheximide (CHX), Lnc-PCIR knockdown accelerate the PABPC4 degradation, whereas Lnc-PCIR activation increased the half-life of the PABPC4 protein in the TNBC cells (**Figure 7A**). Besides, we also treated cells with a proteasome inhibitor MG132, the accumulation of endogenous PABPC4 in cells overexpressing Lnc-PCIR was remarkable greater (**Figure 7B**), indicating that higher level Lnc-PCIR might inhibit the proteasome-dependent degradation of PABPC4 in TNBC cells. Furthermore, the ubiquitination levels of PABPC4 significantly increased in the sh-Lnc-PCIR cells, whereas the ubiquitination levels of PABPC4 sharply decreased in the overexpressed Lnc-PCIR cells (**Figure 7C**). Collectively, these results indicated that Lnc-PCIR can increase the stability of PABPC4 through blocking its ubiquitin/proteasome-dependent degradation.

### TAB3 as the Functional Downstream Mediator of Lnc-PCIR to Activating TNF-α/NF-κB Pathway

Previous studies have shown that TAB2 which tightly associated with TAB3 is involved in NF-κB activation (31). Considering the effect of Lnc-PCIR on TAB3 protein level, we hypothesize that Lnc-PCIR may exert its biological effects through regulating the association of TAB3 and TAB2. The Co-IP study showed that endogenous TAB2, were detected in the immunoprecipitant with TAB3 antibody, and the association of TAB2 and TAB3 was remarkable decreased due to Lnc-PCIR overexpression (**Figure 7D**). Furthermore, functional recovery assay showed deleting TAB3 in the P-Lnc-PCIR cells significantly inhibited the invasion abilities, indicating that TAB3 is an important downstream effector of P-Lnc-PCIR in TNBC cells **(** **Figure 7E**). On the other hand, the activation of NF-κB signaling by treatment with TNFα (10 ng/ml) rescued the decreased cell invasion induced by the knockdown of TAB3. We also treated cells with the NF-κB inhibitor caffeic acid phenethyl ester (CAPE, 10 ng/ml), the transwell assay showed that blockade of NF-κB signaling dramatically decreased Lnc-PCIR induced cell migration and invasion **(** **Figure 7E**). To further clarify the mechanism by which Lnc-PCIR regulates *via* the NF-κB signaling in TNBC cells, we measured the changes in phosphorylated IκBα (p-IκBα), phosphorylated-p65 (p-p65) and total p65 expression in Lnc-PCIR knockdown or overexpression 231 cells. The results showed that the knockdown of Lnc-PCIR can significantly decrease p-IκB and p-p65 in 231 cells and vice-versa **(** **Figure 7F**). TNBC patients in the high TAB3 expression group had a much shorter median survival time than those in the low TAB3 expression group (**Figure 7G**). And TAB3 showed a positive correlation with Lnc-PCIR in TNBC tissues (**Figure 7H**). Taken together, Lnc-PCIR as the oncogenic driver in TNBC, they make a meaningful contribution to tumor progression through activating the NF-κB signaling by associated with TAB3.

## Discussion

Long non-coding RNAs were found to be deregulated in a variety of diseases, especially cancer (32). Understanding the precise molecular mechanism by which lncRNAs function is vital for exploring new potential strategies for early diagnosis and therapy. In this study, from RNA-sequencing data, we identified Lnc-PCIR, is a clinically relevant lncRNA displays a remarkable trend of increased expression in TNBC tissue. Importantly, higher Lnc-PCIR levels predicted lower overall survival rates in TNBC patients, supporting that Lnc-PCIR may be a promising prognostic biomarker for TNBC. Lnc-PCIR showed the strong oncogenic activity by promoting TNBC cell tumor invasion and metastasis, proliferation, tumorigenicity *in vitro* and *in vivo*. And these results may suggest Lnc-PCIR involved in cancer-related biological processes and pathways which lead to TNBC tumorigenesis and progression.

Through the study on the mechanisms driven by Lnc-PCIR, we performed RNA Pulldown and RNA-sequencing assay, and found Lnc-PCIR could directly binds to TAB3 and PABPC4. TAB3 bind to the 5′ terminus of Lnc-PCIR (210–624 nt), whereas the 3’ terminus of Lnc-PCIR (625–987 nt) accounts for its association with PABPC4; therefore, it was not surprising that these two proteins could bind to each other.

TABs family which include TAB1, TAB2 and TAB3 have been identified as the specific binding partner proteins of TAK1 (TGF-β activated kinase 1) which implicated in regulating diverse range of cellular processes that include embryonic development, differentiation, autophagy, apoptosis and cell survival (18, 22). TAB3, a scaffold protein of TAB2, is involved in IL-1 and TNF-α signaling pathways (21). Several reports demonstrated TAB3 is widely expressed and constitutively overexpressed in certain tumor tissues, which as the oncogene to driving the occurrence and development of tumors (24, 33). Here, our findings showed that Lnc-PCIR have a strong impact on TAB3 mRNA stability, and interacting with and upregulating the TAB3 protein level which contribute to the promoting effects on activating TNF-α/NF-κB pathway in TNBC cells. Next, we further explored the mechanisms that stabilize TAB3 mRNA by Lnc-PCIR.

PABPC4, another associated protein of Lnc-PCIR, which has been demonstrated related to the inflammatory biomarker (C-reactive protein) and anti-hepatitis C response, is expressed at a higher level in tumor tissues (34, 35). However, the mechanisms underlying the upregulated protein level of PABPC4 remain unclear. In the present study, we provided a new regulatory mechanism for PABPC4 *via* Lnc-PCIR. Higher level of Lnc-PCIR blocks PABPC4 proteasome-dependent ubiquitination degradation to promote the oncogenic effects of PABPC4 on carcinogenesis of TNBC. Stable and highly expressed PABPC4 can further increase the stability of TAB3 mRNA, and the association of PABPC4 and TAB3 can disrupt the binding of TAB3 and TAB2 to activate the TNF-α/NF-κB pathway.

## Conclusion

This study identifies clinically relevant lncRNA Lnc-PCIR using a large cohort of samples from TCGA and identified it have prognostic value and may be directly implicated in the oncogenic phenotype. Our results show that Lnc-PCIR acts as an oncogene and significantly promotes TNBC cells tumorigenicity and metastasis *in vitro* and *in vivo*. Mechanistically, higher levels of Lnc-PCIR inhibits PABPC4 ubiquitination and degradation. Moreover, higher PABPC4 protein level strengthen the interaction of PABPC4 and TAB3 mRNA, weakens the interaction between TAB3 and TAB2, positively regulated TNF-α/NF-κB signaling pathway. Therefore, our study implies the Lnc-PCIR may provide a potential target for TNBC treatment.

## Data Availability Statement

The original contributions presented in the study are included in the article/**Supplementary Material**. Further inquiries can be directed to the corresponding author.

## Ethics Statement

The studies involving human participants were reviewed and approved by Shanghai Cancer Center of Fudan University. The patients/participants provided their written informed consent to participate in this study. The animal study was reviewed and approved by Fujian Medical University.

## Author Contributions

CW, WG, and HH designed the study. WG, JL, HH, and FF performed the experiments. WG and HH analyzed the results. YL carried out bioinformatics analyses. WG, HH, and CW wrote the paper with comments from all authors. All authors contributed to the article and approved the submitted version.

## Funding

This work was supported by grants from Joint funds for the innovation of science and Technology of Fujian province (2018Y9019, 2018Y9055), Joint funds for the youth research project of Fujian provincial health department (2011-1-14) and minimally invasive medicine center of Fujian Province.

## Conflict of Interest

The authors declare that the research was conducted in the absence of any commercial or financial relationships that could be construed as a potential conflict of interest.

## Abbreviations

LncRNA, Long non-coding RNA; TNBC, Triple Negative Breast Cancer; Lnc-PCIR, LncRNA Positively Correlated with Inflammatory Responses; CCK-8, Cell Counting Kit-8; RIP, RNA Immunoprecipitation; TCGA, The Cancer Genome Atlas; TAB3, TGF-beta activated kinase 1 (MAP3K7) binding protein 3; PABPC4, poly(A) binding protein cytoplasmic 4; TAB2, TGF-beta activated kinase 1 (MAP3K7) binding protein 2; UCSC, University of California, Santa Cruz; qPCR, quantitative real-time PCR; RACE, rapid amplification of cDNA ends; MS, mass spectrometry; ER, estrogen receptor; PR, progesterone receptor; TAK1, Transforming growth factor-β-activated kinase 1; NSCLC, non-small cell lung cancer; HCC, hepatocellular carcinoma; GSEA, Gene Set Enrichment Analysis; GO analysis, Gene Ontology Analysis; EMT, Epithelial-Mesenchymal Transition; CHX, cycloheximide.
