# Supplementary material for: LncRNA PCIR Is an Oncogenic Driver via Strengthen the Binding of TAB3 and PABPC4 in Triple Negative Breast Cancer
Source: Front Oncol. 2021 May 3;11:630300. doi: 10.3389/fonc.2021.630300 (PMC8128249; doi:10.3389/fonc.2021.630300)
Supplement: Supplementary file 3 [file DataSheet_3.docx]

**Supplementary material**

**LncRNA PCIR is an Oncogenic Driver via Strength the Binding of TAB3 and PABPC4 in Triple Negative Breast Cancer**

Wenhui Guo^1,2,3,*^, Jingyi Li^4,*^, Haobo Huang^5,*^, Fangmeng Fu^1,2,3*^, Yuxiang Lin^1,2,3^, Chuan Wang^1,2,3,#^.

^1^Department of Breast Surgery, Fujian Medical University Union Hospital, Fuzhou, Fujian Province, 350001, China;

^2^Department of General Surgery, Fujian Medical University Union Hospital, Fuzhou, Fujian Province, 350001, China;

^3^Breast Cancer Institute, Fujian Medical University, Fuzhou, Fujian Province, 350001, China;

^4^College of Integrated Traditional Chinese and Western Medicine, Fujian University of Traditional Chinese Medicine, Fuzhou, Fujian Province, 350122, China;

^5^Department of blood transfusion, Fujian Medical University Union Hospital, Fuzhou, Fujian Province, 350001, China.

*These authors contribute equally to this work

**^#^Co-corresponding author**.

Chuan Wang, Email: chuanwangfmu@163.com

Address: No.29, Xinquan Road, Gulou District, Fuzhou City, Fujian Province, China

Tel: (86)-591-83357896; Fax: (86)-591-87113828

**Supplementary Figures**


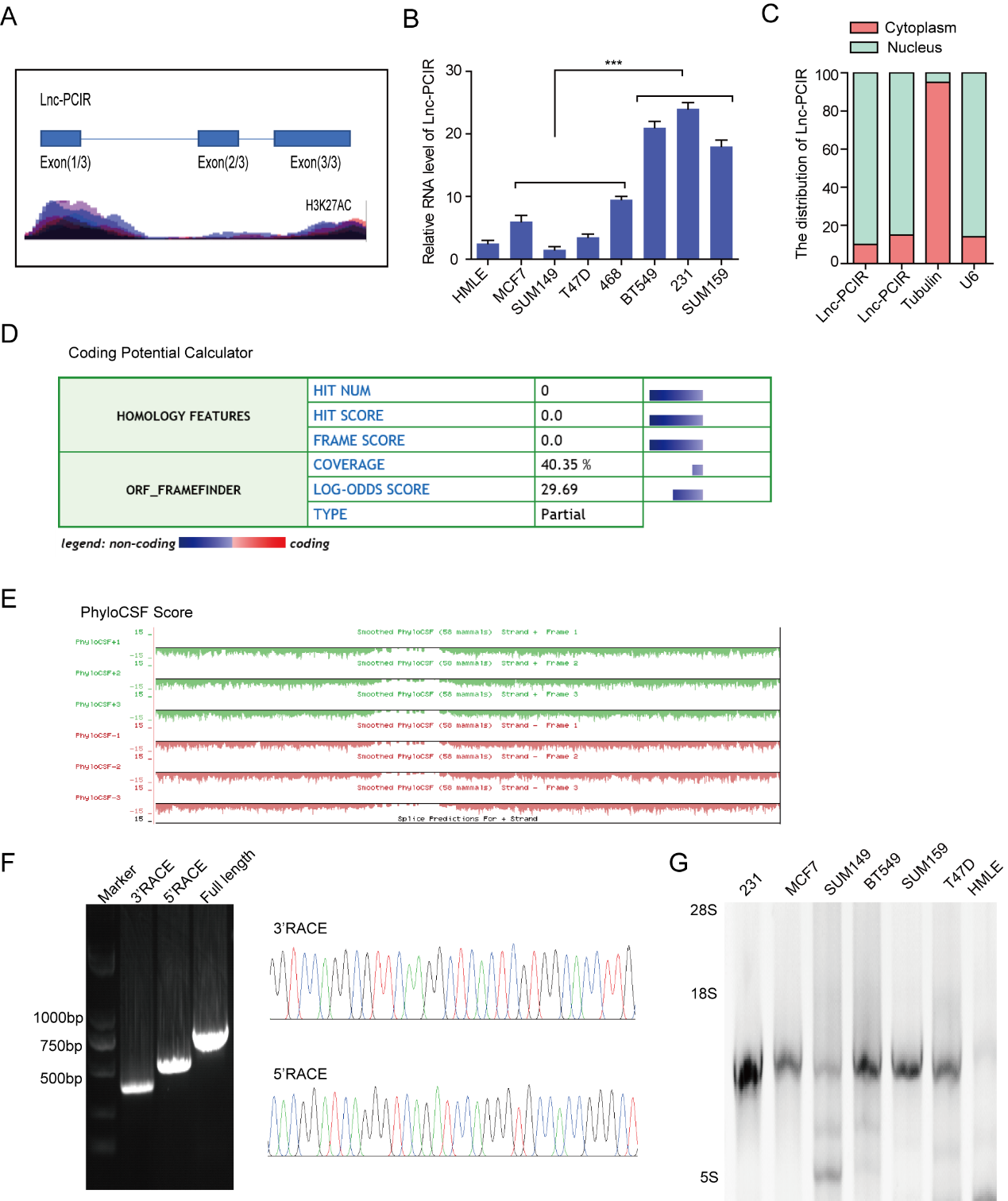


Fig S1: Basic characteristics of Lnc-PCIR in TNBC cells

A: Schematic representation of exons and transcripts of Lnc-PCIR in UCSC Genome browser (GRCh37/hg38). B: The background RNA level of Lnc-PCIR in several TNBC cell lines. C: The distribution of Lnc-PCIR RNA in 231 and BT549 cells. D, E: Predicted the protein-coding potential of Lnc-PCIR by Coding Potential Calculator (D) and PhyloCSF software (E). F: Representative image of PCR products from the 3’ and 5’ RACE and full-length of Lnc-PCIR in 231 cells. G: Northern-blot verified the full-length of Lnc-PCIR in seven TNBC cell lines.


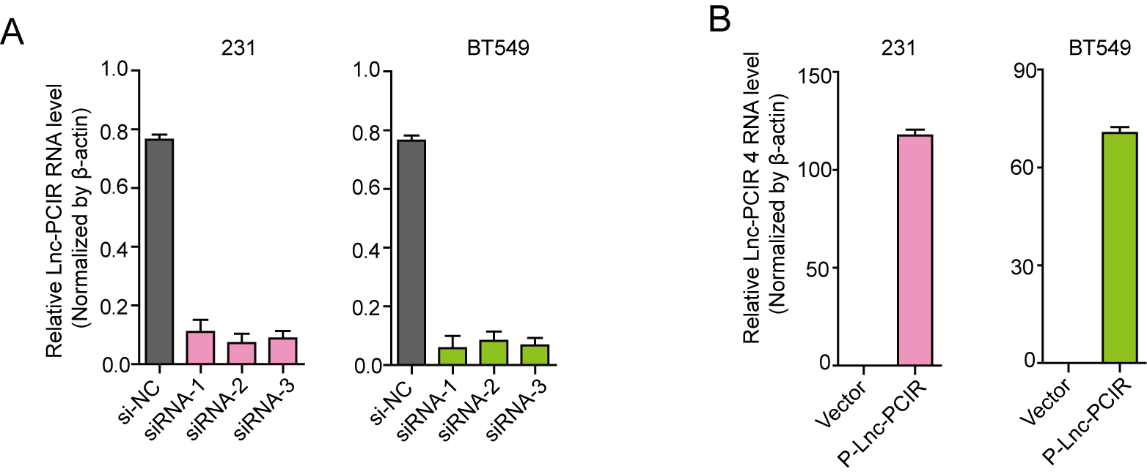


Fig S2: The efficiency of siRNAs and stable overexpressed Lnc-PCIR

A: The efficiencies of three independent siRNAs for Lnc-PCIR in 231 and BT549 cells. B: The overexpression of Lnc-PCIR by lentivirus in 231 and BT549 cells.


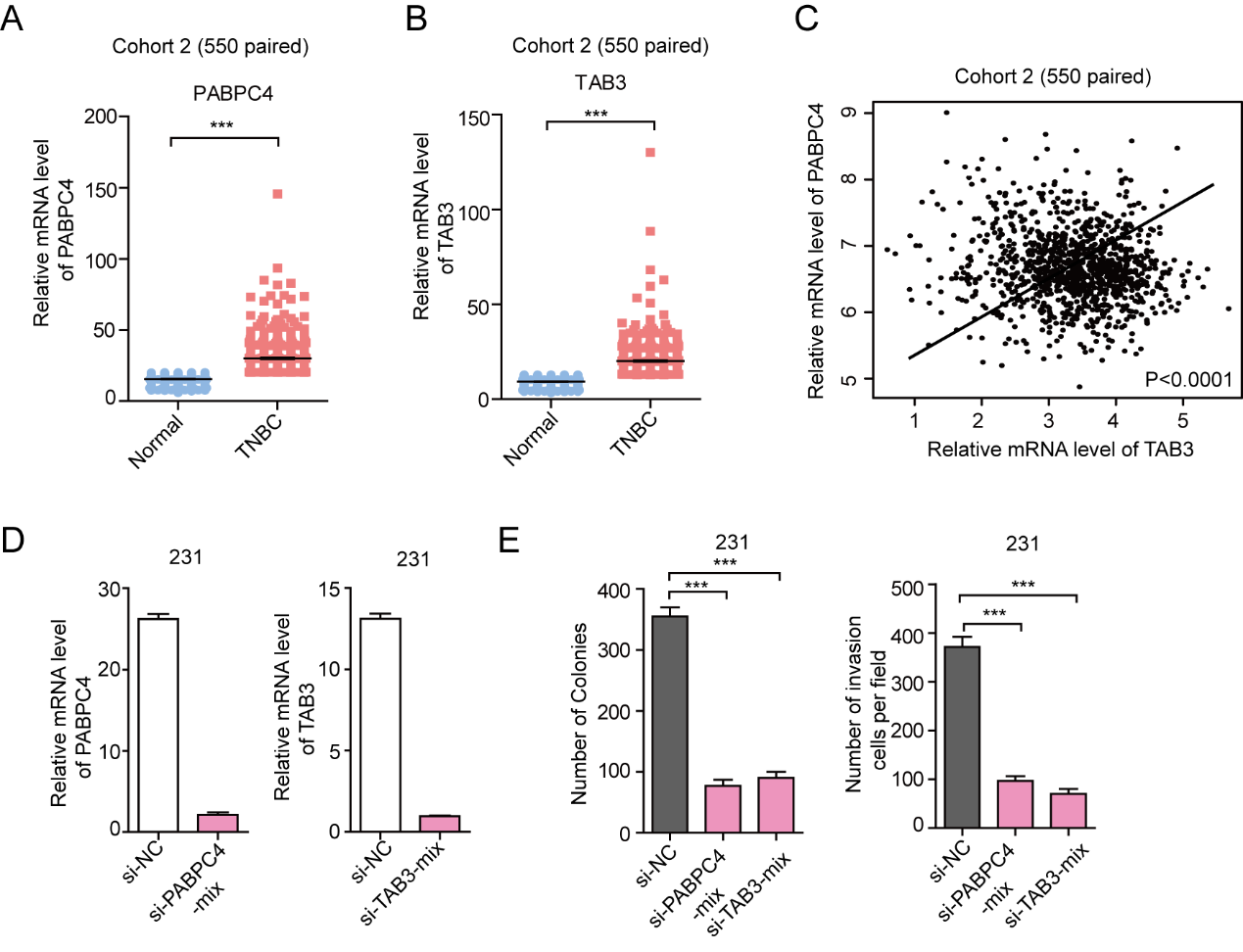


Fig S3: Both PABPC4 and TAB3 as the oncogenes in TNBC cells.

A, B: The RNA levels of PABPC4 and TAB3 were analysed in 550 paired TCGA TNBC and adjacent non-tumour samples. C: PABPC4 had a positive relation with TAB3 in in 550 paired TCGA TNBC. D: The efficiencies of knockdown of PABPC4 and TAB3 mix with two independent siRNAs in 231 cells. E: Colony formation assays and Transwell invasion assays performed when knockdown of PABPC4 or TAB3.


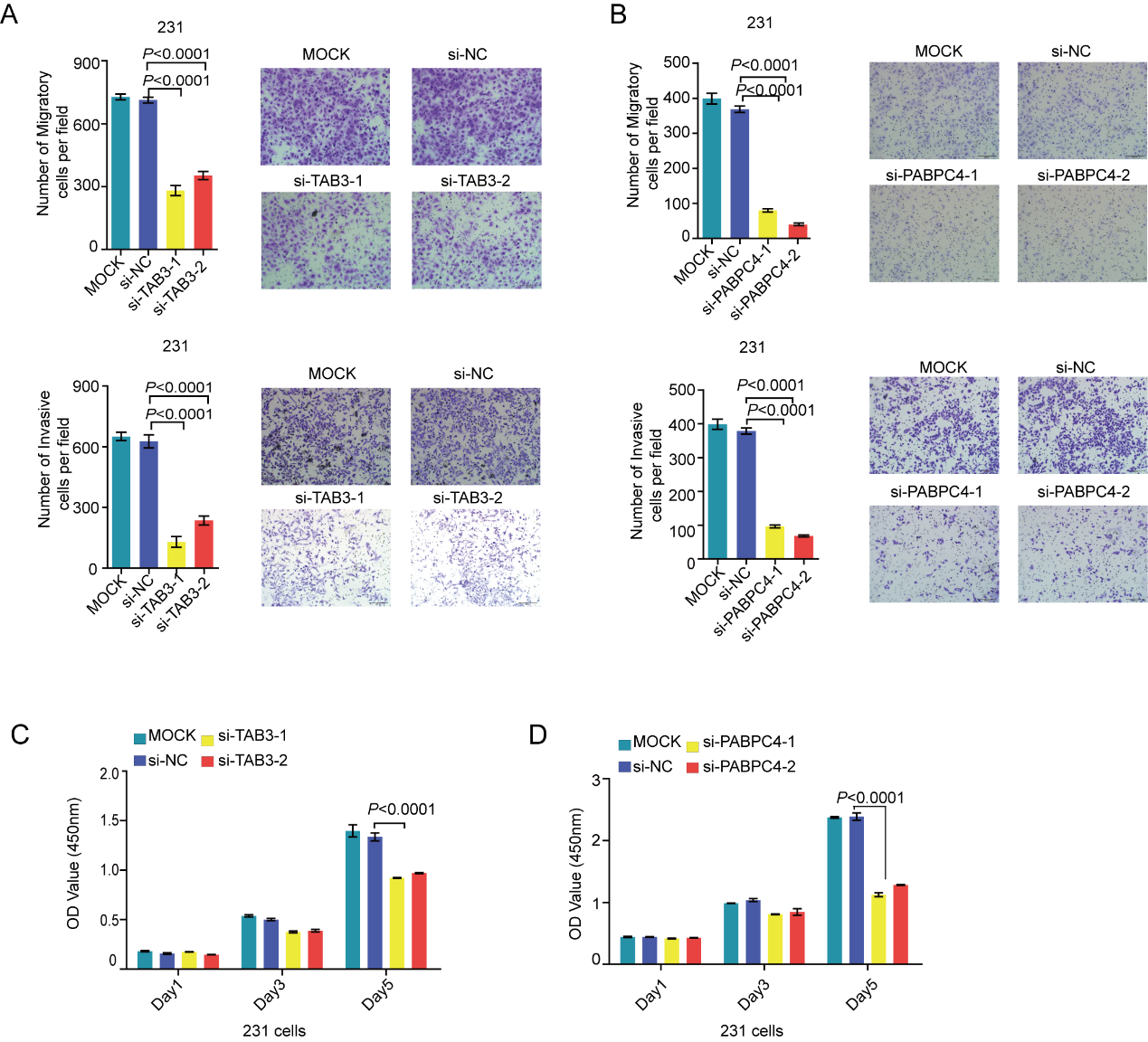


Fig S4: Knockdown of Lnc-PCIR decreases 231 cells proliferation, invasion and metastasis *in vitro*.

A. Transwell migration and invasion assays in the 231 cells with TAB3 knockdown; B: Transwell migration and invasion assays in the 231 cells with PABPC4 knockdown; C, D. CCK-8 assays in the 231 cells with knockdown of TAB3 (C) and PABPC4 (D).

**Supplementary Tables**

Supplementary Table 1. Relationships between Lnc-PCIR expression and clinicopathologic factors of the patients with TNBC

| Characteristics |  | Low | High | p-value |
| --- | --- | --- | --- | --- |
| Age | <40 | 6 | 8 | 0.83 |
|  | ≥40 | 40 | 56 |  |
| Tumor grade | Ⅰ&Ⅱ | 20 | 25 | 0.56 |
|  | Ⅲ | 35 | 40 |  |
| pT | pT1 | 15 | 18 | 0.42 |
|  | pT2 | 35 | 30 |  |
|  | pT3 | 7 | 5 |  |
| pN | pN0 | 13 | 15 | 0.57 |
|  | pN1 | 17 | 19 |  |
|  | pN2 | 18 | 20 |  |
|  | pN3 | 4 | 7 |  |
| LV1 | Negative | 10 | 15 | 0.49 |
|  | Positive | 35 | 50 |  |

Abbreviations: LVI: lymphovascular invasion. Fisher exact test was used.

**Supplementary Table 2**. Primer, probe and siRNAs sequence used in this study

| Primer |  | Sequence |
| --- | --- | --- |
| Lnc-PCIR-F |  | GCTGGTAAGAGCGTAGGTGAA |
| Lnc-PCIR-R |  | GCTTCTTACTGCGGCTTCAT |
| PABPC4-R |  | GCTCCCTGGGCTATGCCTACGT |
| PABPC5-F |  | TCCCGCTCTTTGCGAGACTTGA |
| TAB3-F |  | TCACCCATCAGTAATCAACCAT |
| TAB3-R |  | ACTTCAGACTTCAACCGCTCTA |
| U6-F |  | CTCGCTTCGGCAGCACATAT |
| U6-R |  | TATGGAACGCTTCACGAATTTG |
| GAPDH-F |  | AACGGGAAGCTTGTCATCAA |
| GAPDH-R |  | TGGACTCCACGACGTACTCA |
| ACTB-F |  | GTTGTCGACGACGAGCG |
| ACTB-R |  | GCACAGAGCCTCGCCTT |
| TAB3-pcDNA-F |  | CGGGATCC ATGGCGCAAAGCAGCCCACAGCTTG |
| TAB3-pcDNA-R |  | CGGAATTC TCAGGTGTACCGTGGCATCTCGCAC |
| PABPC4-pcDNA-F |  | GGGGTACCCC ATGAACGCTGCGGCCAGCAGCTACC |
| PABPC4-pcDNA-R |  | CCAAGCTTGG CTAAGAGGTAGCAGCAGCAACAGCG |
| Lnc-PCIR-Northern-F |  | GGAAAAGATCACTAAAAAGCTTCTC |
| Lnc-PCIR-Northern-R |  | CCACCAGGTTTGCGTAATGGGCTGA |
| Lnc-PCIR-3'RACE |  | CTCAGCCTGCTCCCAACCAGGTGTCTC |
| Lnc-PCIR-5'RACE |  | GAGACACCTGGTTGGGAGCAGGCTGAG |
| MYC-F |  | AGCTGCTTAGACGCTGGATTT |
| MYC-R |  | CGAGGTCATAGTTCCTGTTGGT |
| LOC645249-F |  | CCTGCGCATGCGTGATACTTGCACC |
| LOC645249-R |  | CGAGTTGTATGTTGTCTGCGTTGTT |
| SNHG3-F |  | GATTCTCTAACTGCGCATGCTTCTG |
| SNHG3-R |  | AGGAGAAGAAGCGACCA |
| LINC00160-F |  | GAATGAGGAAGCTCTCTGTG |
| LINC00160-R |  | AACCCTGGAAATCTTGTG |
| BCL-XL-F |  | GAATCTCTTTCTCTCCCTTCAG |
| BCL-XL-R |  | AAACTCGTCGCCTGCCTCCCTC |
| Survivin-F |  | 5' CAAGGACCACCGCATCT 3' |
| Survivin-R |  | 5' CACCAAGGCACCAGCAT 3' |
| VCAM1-F |  | GAGCGGGAAGGTGAGGA |
| VCAM1-R |  | GAAGGGCTGACCAAGACG |
| Cox2-F |  | TCCCATCCCTACGCATCC |
| Cox2-R |  | CGGTCGTGTAGCGGTGAA |
| Cyclin D1-F |  | GAACAGAAGTGCGAGGAGGAG |
| Cyclin D1-R |  | AGGCGGTAGTAGGACAGGAAG |
| IL-8-F |  | TGGCAGCCTTCCTGATT |
| IL-8-R |  | ACTTCTCCACAACCCTCT |
| CD44-F |  | CAGACAGAATCCCTGCTAC |
| CD44-R |  | TCTCCGTTGAGTCCACTT |
| MMP9-F |  | TCCCTGGAGACCTGAGAACC |
| MMP9-R |  | CGGCAAGTCTTCCGAGTAGTTT |
| Lnc-PCIR-full length-F |  | TAATACGACTCACTATAGGGAGA CCTGCGCATGCGTGATACTTGCACC |
| Lnc-PCIR-full length-R |  | TTTTCCAAGGATTTATTTAATGGGG |
| Lnc-PCIR-#1-F |  | TAATACGACTCACTATAGGGAGA CCTGCGCATGCGTGATACTTGCACC |
| Lnc-PCIR-#1-R |  | TGCGCTCGTAAGCTGCGCCCGGCGC |
| Lnc-PCIR-#2-F |  | TAATACGACTCACTATAGGGAGA TCTCCGAGTATGAAAGTGCGAGGAG |
| Lnc-PCIR-#2-R |  | TTCTGGAGACACCTGGTTGGGAGCA |
| Lnc-PCIR-#3-F |  | CCTGGAAATCTGGATTGAGC CCTAGACCCCCGCTCGGGCCACGCG |
| Lnc-PCIR-#3-R |  | TTTTCCAAGGATTTATTTAATGGGG |
| si-Lnc-PCIR-1 |  | CCTTGGGAATCCGAGGAAT |
| si-Lnc-PCIR-2 |  | GCTCAGCCCATTACGCAAA |
| si-Lnc-PCIR-3 |  | GGTCTTGCCTTGAACACAT |

**Supplementary Table 3. Antibodies for western-blot, RIP and IP**

| Protein Name | Company | Catalog Number | Dilutions in WB | Dilutions in RIP/IP |
| --- | --- | --- | --- | --- |
| TAB3 | Cell Signaling Technology | #14241 | 1:1000 | 5μg |
| PABPC4 | Abgent | A-AP17240c | 1:1000 | 5μg |
| Ubiquitin Antibody | Proteintech | 10201-2-AP | 1:1000 | N/A |
| IKBα | Cell Signaling Technology | #4814 | 1:1000 | N/A |
| p-IKBα | Cell Signaling Technology | #8219 | 1:5000 | N/A |
| P65 | Cell Signaling Technology | #8242 | 1:5000 | N/A |
| p-p65 | Cell Signaling Technology | #3033 | 1: 5000 | N/A |
| GAPDH | Proteintech | 60004-1-IG | 1:2000 | 5μg |

**Supplementary Table 4.** Mass Spectrometry protein identification results for biotinylated Lnc-PCIR RNA pull-down assay

|  | **Lnc-PCIR-Sense** | | | | | | **Lnc-PCIR-Antisense** | | | | | |
| --- | --- | --- | --- | --- | --- | --- | --- | --- | --- | --- | --- | --- |
| **Protein Name** | **Unique Peptides** | | | **Peptides** | | | **Unique Peptides** | | | **Peptides** | | |
|  | **#1** | **#2** | **#3** | **#1** | **#2** | **#3** | **#1** | **#2** | **#3** | **#1** | **#2** | **#3** |
| TGF-beta activated kinase 1 binding protein 3 | 15 | 15 | 15 | 15 | 15 | 15 | NA | NA | NA | NA | NA | NA |
| TGF-beta activated kinase 1 binding protein 2 | 10 | 10 | 10 | 14 | 14 | 14 | NA | NA | NA | NA | NA | NA |
| IKB Kinase-Associated protein 1 | 10 | 10 | 10 | 14 | 14 | 14 | NA | NA | NA | NA | NA | NA |
| TNF Receptor-Associated Factor 6 | 10 | 10 | 10 | 11 | 11 | 11 | NA | NA | NA | NA | NA | NA |
| Interferon alpha 1 | 9 | 9 | 9 | 10 | 10 | 10 | NA | NA | NA | NA | NA | NA |
| Eukaryotic translation initiation factor 5B | 6 | 6 | 6 | 10 | 10 | 10 | NA | NA | NA | NA | NA | NA |
| Poly(A)-Binding Protein, Cytoplasmic4 | 6 | 6 | 6 | 10 | 10 | 10 | NA | NA | NA | NA | NA | NA |
| RecQ Protein-Like 1 | 5 | 5 | 5 | 10 | 10 | 10 | NA | NA | NA | NA | NA | NA |
| Toll Like Receptor 4 | 5 | 5 | 5 | 10 | 10 | 10 | NA | NA | NA | NA | NA | NA |
